# Early detection of acute coronary syndrome using traditional versus emerging biochemical markers

**DOI:** 10.6026/973206300210870

**Published:** 2025-04-30

**Authors:** Minakshi Kumari, Saket Kumar, Jhuma Das

**Affiliations:** 1 Department of Biochemistry, Netaji Subhas Medical College and Hospital, Jamshedpur, Jharkhand, India; 2 Department of Pathology, Netaji Subhas Medical College, Jamshedpur, Jharkhand, India

**Keywords:** Acute coronary syndrome, myocardial injury, troponin I, creatine kinase-MB (CK-MB), heart-type fatty acid-binding protein (H-FABP), copeptin, ischemia-modified albumin, early diagnosis

## Abstract

Early detection of acute coronary syndrome (ACS) becomes more accurate using dependable biomarkers for diagnosis. Therefore, it is of interest to evaluate using established markers (Troponin I, CK-MB) together with emerging markers (H-FABP, Copeptin, IMA) for 150 patients. Analysis of H-FABP yielded the best early sensitivity rate at 92% surpassing both Troponin I and CK-MB values. The combination of H-FABP with Troponin I produced superior ACS detection results (p < 0.05). The use of emerging biomarkers shows better diagnostic precision for standard practice in ACS testing.

## Background:

Acute coronary syndrome (ACS) contains unstable angina together with non-ST-elevation myocardial infarction (NSTEMI) and ST-elevation myocardial infarction (STEMI) as its clinical conditions when an atherosclerotic plaque rupture and subsequent thrombus formation produce myocardial ischemia [1]. Early detection together with precise diagnosis of myocardial injury serves as a main requirement to initiate proper treatments that can enhance patient outcomes. The use of biomarkers serves essential functions for diagnosing ACS and risk appraisal as well as forecasting patient outcomes [2]. Research shows that cardiac troponins (cTnI and cTnT) represent the best method for myocardial injury detection because of their exceptional ability to detect myocardial necrosis [3]. Troponins need several hours for their concentration to increase following myocardial infarction making them insufficient for early ACS identification [4]. Two historical myocardial injury markers creatine kinase-MB (CK-MB) and Myoglobin provide limited diagnostic value because they do not satisfy the requirements for definitive diagnosis of ACS [5]. Research activities now aim to identify new biomarkers which improve the detection of myocardial injuries in an early stage. Heart-type fatty acid-binding protein (H-FABP) functions as a rapidly released cytoplasmic protein which researchers believe shows promise as an initial biomarker of ACS [6]. The stress-induced hormone fragment Copeptin retains its stability to provide additional diagnostic help when used with troponins in early ACS evaluation [7]. Ischemia-modified albumin (IMA) serves as an emerging diagnostic tool because it tracks myocardial ischemic activity without demonstrating necrotic properties thus becoming beneficial for patient risk identification that precedes troponin gradient increase [8]. Novel biomarkers such as MPO, IMA, hs-CRP, and microRNAs enhance early detection and risk assessment of acute coronary syndrome beyond traditional troponins [9]. Therefore, it is of interest to investigate the diagnostic capabilities of standard markers Troponins, CK-MB, Myoglobin compared to contemporary biomarkers H-FABP, Copeptin, IMA when used for ACS early detection.

## Materials and Methods:

The study conducted at a tertiary care hospital performed prospective observations on patients exhibiting chest pain signs for acute coronary syndrome (ACS). The study included 150 patients between 30 and 75 years old who showed ACS symptomatology within the first six hours after symptoms started. Chronic renal disease patients together with individuals suffering from liver disorders or recent trauma received exclusion from study participation due to biomarker level interference. Scientists obtained venous blood specimens through three different collection periods starting from admission time (0 hours) continuing to 3 hours and finally to 6 hours after patient admission. The serum specimens were cooled after centrifugation before storing them at -80°C until evaluation.

## Traditional biomarkers:

The laboratory performed chemical immunoassays for both troponin I (cTnI) and Creatine Kinase-MB (CK-MB) measurements. Testing of Myoglobin biomarkers occurred through enzyme-linked immunosorbent assay (ELISA).

## Novel biomarkers:

The determination of heart-type fatty acid-binding protein (H-FABP) levels occurred through an ELISA kit method. The electrochemiluminescence immunoassay method determined levels of Copeptin which operates as a stable indicator of stress response. The analysis of Ischemia-Modified Albumin (IMA) occurred through the albumin cobalt-binding test.

## Clinical and diagnostic criteria:

The analysis group included patients who met criteria comprised of both clinical presentation and electrocardiographic results and indications of biomarker increase. Cardio-specialist analyzed ECG readings independently to verify which patients had ACS.

## Statistical analysis:

Data analysis occurred through SPSS software version 25.0. The analysis displayed continuous variables through mean ± standard deviation (SD) while independent t-test or ANOVA conducted variable comparisons. Categorical variables received statistical interpretation through chi-square testing while showing their results as percentages. The clinical value of biomarkers was evaluated among the parameters that included sensitivity and specificity and positive predictive value (PPV) and negative predictive value (NPV). The diagnostic comparison between conventional and new biomarkers was conducted through Receiver Operating Characteristic (ROC) curves which produced area under curve (AUC) assessments. Results with p-value less than 0.05 were counted as statistically significant for this study.

## Results:

The research analyzed 150 participants whose average age amounted to 58.4 ± 9.6 years. The patient group consisted of 90 males who made up 60% of the total while female participants accounted for 40% with 60 individuals. The study participants showed hypertension in 72 patients (48%) while diabetes mellitus occurred in 58 (38.7%) and 65 individuals (43.3%) had smoking habits. Table 1 presents an overview of the fundamental traits that characterize the patients enrolled in the study. At 3 hours the levels of Troponin I reached elevated levels in 93 (62%) patients who also demonstrated an increase in the levels of CK-MB in 85 (56.7%) patients. Early detection capabilities were indicated by novel biomarker results where H-FABP levels increased in 110 (73.3%) patients and Copeptin levels increased in 108 (72%) patients and also IMA levels increased in 112 (74.7%) patients. The evaluation of biomarker performance is presented in full detail through Table 2. H-FABP demonstrated the highest sensitivity (92%), followed by IMA (91%) and Copeptin (89%), compared to Troponin I (85%) and CK-MB (75%). Novel biomarkers also showed better negative predictive values, suggesting their utility in early ACS exclusion (Table 2). ROC analysis revealed that H-FABP had the highest area under the curve (AUC = 0.94), followed by IMA (AUC = 0.92) and Copeptin (AUC = 0.91). Troponin I exhibited an AUC of 0.89, while CK-MB had an AUC of 0.82. The detailed ROC values are shown in Table 3. The combined use of Troponin I with H-FABP significantly improved early detection rates, highlighting the advantage of integrating novel biomarkers with traditional ones for ACS diagnosis (Table 3).

## Discussion:

Medical personnel need to detect acute coronary syndrome (ACS) as early as possible and with high specificity because such detection enables immediate medical intervention that leads to better patient recovery. The three commonly used biomarkers Troponin I, CK-MB and Myoglobin show delayed elevations following myocardial injury so their effectiveness diminishes in early ACS detection [1]. The research findings showed heart-type fatty acid-binding protein (H-FABP), copeptin and ischemia modified albumin (IMA) exceeded traditional biomarkers when detecting acute coronary syndrome diagnosis at an early stage. Medical research has established Troponin I as the preferred diagnostic marker for ACS because it specifically detects myocardial injuries [2]. However, its delayed release, typically 3-6 hours after symptom onset, poses a challenge for early detection [3]. The results of Troponin I demonstration an 85% sensitivity and 90% specificity in this assessment while meeting the findings of previous research regarding myocardial infarction detection [4]. The diagnostic accuracy of CK-MB and Myoglobin was found to be inferior to troponins based on previous research findings as well as the study results [5, 6]. Novel biomarker H-FABP achieved 92% sensitivity as well as 88% specificity in this study. The release of H-FABP into circulation soon after myocardial damage enables its use as an early warning sign for ACS because it belongs to a group of proteins with low molecular weight [7]. The research community continues to record comparable results which highlight H-FABP potential application in second stage ACS detection [8]. Research has indicated that Copeptin maintains 89% sensitivity and 87% specificity to support its early use in diagnosing acute coronary syndrome [10]. The rapid increase of Copeptin levels after stress or myocardial ischemia shows that it works well together with Troponin I testing [11]. Multiple research findings indicate improved diagnostic precision occurs when healthcare providers measure Copeptin together with Troponin I particularly for patients having non-detectable troponin levels even though they display ACS symptoms [12]. Research findings demonstrated that IMA exhibits 91% sensitivity together with 86% specificity as an emerging biomarker. The formation of IMA occurs because of oxidative stress and ischemic situations that make this biomarker a trustworthy indicator for detecting initial myocardial ischemia [13]. Numerous studies have demonstrated how IMA helps medical professionals identify myocardial ischemia among other causes of chest pain [14]. Research findings in this study confirm previous studies about IMA operating as a fast diagnostic indicator. The analysis with receiver operating characteristic (ROC) determined H-FABP demonstrated the highest area under the curve (AUC = 0.94) compared to IMA with AUC = 0.92 and Copeptin with AUC = 0.91. Multiple previous meta-analyses prove that these new biomarkers perform better than traditional markers when used alone [15]. The integration of H-FABP with Troponin I led to better early diagnosis rates thus demonstrating the value of routine clinical practice with new biomarkers [17]. Novel biomarkers such as cMyC, IMA, microRNAs, and copeptin enhance early diagnosis, monitoring, and prognosis of acute myocardial infarction beyond traditional markers [17]. The study has some limitations. Generalization of research findings is restricted because this study utilized a small participant count conducted at a single institution. This research does not include follow-up data to provide information about long-term prognostic results. The clinical benefit of these emerging biomarkers demands additional multi-site tests to establish proper application standards for regular ACS diagnosis.

## Conclusion:

The early diagnosis of ACS becomes more sensitive when physicians use H-FABP, Copeptin and IMA biomarkers above traditional biomarkers. The diagnostic precision for ACS will improve if physicians include these biomarkers along with Troponin I to begin proper treatment without delay. Hence, their clinical usage must be increased to create guidelines for standard implementation as diagnostic protocol across routine care.

## Figures and Tables

**Table 1:** Baseline demographic and clinical characteristics of patients

**Characteristic**	**Value (n = 150)**
Mean Age (years)	58.4 ± 9.6
Male (%)	90 (60%)
Female (%)	60 (40%)
Hypertension (%)	72 (48%)
Diabetes Mellitus (%)	58 (38.7%)
Smoking History (%)	65 (43.3%)

**Table 2:** Comparison of biomarker sensitivity and specificity in ACS diagnosis

**Biomarker**	**Sensitivity (%)**	**Specificity (%)**	**PPV (%)**	**NPV (%)**
Troponin I	85	90	88	87
CK-MB	75	85	80	82
Myoglobin	70	78	74	76
H-FABP	92	88	90	91
Copeptin	89	87	88	89
IMA	91	86	89	90

**Table 3:** ROC curve analysis for biomarker performance in ACS detection

**Biomarker**	**Area Under Curve (AUC)**
Troponin I	0.89
CK-MB	0.82
Myoglobin	0.79
H-FABP	0.94
Copeptin	0.91
IMA	0.92
